# The Pbx Interaction Motif of Hoxa1 Is Essential for Its Oncogenic Activity

**DOI:** 10.1371/journal.pone.0025247

**Published:** 2011-09-21

**Authors:** Stéphanie Delval, Arnaud Taminiau, Juliette Lamy, Cécile Lallemand, Christine Gilles, Agnès Noël, René Rezsohazy

**Affiliations:** 1 Molecular and Cellular Animal Embryology Group, Life Sciences Institute (ISV), Université Catholique de Louvain, Louvain-la-Neuve, Belgium; 2 Laboratory of Biology of Tumors and Development, GIGA-Cancer, University of Liège and Centre Hospitalier Universitaire, Liège, Belgium; Wellcome Trust Centre for Stem Cell Research, United Kingdom

## Abstract

Hoxa1 belongs to the Hox family of homeodomain transcription factors involved in patterning embryonic territories and governing organogenetic processes. In addition to its developmental functions, *Hoxa1* has been shown to be an oncogene and to be overexpressed in the mammary gland in response to a deregulation of the autocrine growth hormone. It has therefore been suggested that Hoxa1 plays a pivotal role in the process linking autocrine growth hormone misregulation and mammary carcinogenesis. Like most Hox proteins, Hoxa1 can interact with Pbx proteins. This interaction relies on a Hox hexapeptidic sequence centred on conserved Tryptophan and Methionine residues. To address the importance of the Hox-Pbx interaction for the oncogenic activity of Hoxa1, we characterized here the properties of a Hoxa1 variant with substituted residues in the hexapeptide and demonstrate that the Hoxa1 mutant lost its ability to stimulate cell proliferation, anchorage-independent cell growth, and loss of contact inhibition. Therefore, the hexapeptide motif of Hoxa1 is required to confer its oncogenic activity, supporting the view that this activity relies on the ability of Hoxa1 to interact with Pbx.

## Introduction

*Hox* genes define a subset of the homeobox gene family coding for homeodomain transcription factors involved in mammalian embryogenesis and organogenesis [1], [2], [3]. They contribute to pattern the main body axis and the limbs and they control cell fate determination in several organs and cell lineages [4], [5], [6]. Misregulation of *Hox* genes has been reported to be associated with the development of a variety of human cancers, including those of skin [7], breast [8], lung [9], prostate, and blood cells [10]. Whether this association between tumorigenesis and *Hox* gene misexpression reveals that *Hox* genes actually contribute to the transformation process, is an issue that remains largely unresolved. Only a few Hox proteins have actually been proved to act on cancer progression, either as oncoproteins or tumor suppressors [11], [12], [13].

In the normal mammary gland, distinct *Hox* genes exhibit specific expression patterns and functions along its successive development phases, from prenatal stages to lactation at adulthood [14]. *Hoxc6* is expressed during mammary development and this expression declines during pregnancy [15] while *Hoxa9*, *Hoxb9* and *Hoxd9* are required for the expansion and/or differentiation of the mammary epithelial ductal system in response to pregnancy [16] and targetted disruption of *Hoxd10*, leads to a failure in alveolar expansion in late pregnancy and concomitant lactation defect [17].

In addition to their involvement in the normal mammary gland biology, studies have shown that some *Hox* genes are repressed or overexpressed in mammary carcinomas and therefore influence cancer progression. For example, when *HOXA10* is expressed in both benign and malignant breast tissue in adult women, it impacts on tumor cell phenotype by decreasing cell invasiveness and upregulating the tumor suppressor gene p53 [18]. HOXA5 is also a positive regulator of p53 in the normal breast tissue. In human breast tumors, p53 expression can be dramatically decreased by a compromised *HOXA5* function [19], and expression of *HOXA5* in epithelial cancer cells displaying wild-type p53 led to apoptotic cell death. *HOXD10* also has a tumor suppressor function. Its expression is progressively reduced in epithelial cells as malignancy increases in breast tumors and restored *Hoxd10* activity inhibits tumor development in mouse xenografts and impairs migration of tumor cells [20]. HOXA9 positively regulates BRCA1 expression and represses breast tumor growth and malignancy [21]. While several Hox proteins act as tumor suppressors, *HOXB7* is overexpressed in primary breast carcinoma and metastasis, and it stimulates tumor progression by promoting epithelial-mesenchymal transition [22].

*Hoxa1* is one of the first *Hox* genes to be expressed during embryonic development [23]. Gene inactivation has demonstrated its functional importance for hindbrain segmentation, hindbrain patterning, inner and middle ear organogenesis and skull basis morphogenesis [24]. While Hoxa1 is not expressed in the adult mammary gland, several studies revealed that it can be upregulated in mammary carcinomas [8], [15], [17], [25]. Hoxa1 can be activated in mammary epithelial cells in response to an increased autocrine growth hormone (hGH) stimulation which leads to cell transformation as well as cancer progression and invasiveness [26], [27], [28]. Forced expression of Hoxa1 is sufficient to provoke the oncogenic transformation of immortalized human mammary epithelial cells and formation of tumors *in vivo* after cell grafting in mice [29].

Several Hoxa1 target genes have been identified to take part in carcinogenesis. Genes coding for signal tranducing proteins active in the p44/42 mitogen-activated protein (MAP) kinase pathway (GRB2, MEK1, SDFR1) are downstream targets of Hoxa1 [30]. Some p44/42 MAP kinase-regulated genes (IER3, EPAS1, PCNA, catalase) can also be modulated by Hoxa1 [30]. Hoxa1 has further been demonstrated to stimulate oncogenicity by activating *STAT3*, *STAT5B* [31] and the anti-apoptotic gene *BCL-2*, with the consequence to dramatically reduce the apoptotic cell death [29]. Another gene directly regulated by Hoxa1, *EphA2*, has also been reported to transform mammary epithelial cells and to promote tumor formation *in vivo* [32]. Expression of EphA2 and its ligand ephrin-A1 has been observed in the vasculature of human primary breast cancer and of breast-tumor-cell-line-derived tumors in nude mice. Thus, EphA2 has been proposed to be involved in tumor-induced angiogenesis [33]. Furthermore, Hoxa1 promotes the activation of Cyclin-D1 required for the autocrine hGH-mediated cell cycling stimulation in mammary carcinoma [29], [34]. Finally, an increased *Hoxa1* expression is not only observed upon autocrine hGH stimulation but can also occur as a consequence of E-cadherin-mediated signalling. Hoxa1 activation is required for E-cadherin-dependent anchorage-independent proliferation and decreases apoptotic cell death of human mammary carcinoma cells [35].

As transcription factors, Hox proteins cooperate with other transcription regulators or coregulators [36], [37], [38], [39]. Such interactions affect the DNA binding specificity and/or the transcriptional activity of the Hox proteins [40], [41], [42], [43], [44]. Among the best characterized Hox cofactors are the Three-Amino-acid-Loop-Extension (TALE) family of homeodomain proteins [45], [46], which can be subdivided into four groups according to sequence similarities: PBC (*Pbx*, *ceh-20*, *exd*), TGIF, MEIS (*Meis*, *ceh-25*, *hth*, *Prep*) and IRO [47], [48]. The Pbx proteins belong to the PBC group of TALE proteins able to cooperatively bind to DNA with Hox proteins of paralogy groups 1–10. *In vitro* studies have shown that Hox/Pbx heterodimers display a greater affinity and specificity for cognate DNA sequences than the Hox monomers [41], [49]. The interaction between Hox proteins of paralog groups 1–8 and Pbx relies on a conserved hexapeptide sequence located N-terminal to the Hox homeodomain and sharing core Tryptophan and Methionine residues. Hox proteins of paralog groups 9 and 10 do not contain this hexapeptide, they only present a conserved Tryptophan allowing their interaction with Pbx [50], [51], [52], [53].

Mutational analysis of Hoxa1 has revealed that the Tryptophan and Methionine residues of the conserved hexapeptide are critical for the cooperative interaction between Hoxa1 and Pbx1 [42]. Moreover, the mutant Hoxa1 protein was found to be inactive on cognate target enhancers in live cells [54]. Finally, *in vivo* studies have demonstrated that knock-in mice for mutations resulting in a WM-to-AA substitution in the hexapeptide of Hoxa1 display hindbrain, cranial nerve and skeletal defects corresponding to the phenotype of the Hoxa1 knock-out [55]. Together, these data support that the embryonic function of Hoxa1 requires the integrity of its hexapeptide motif, which in turn suggests that the activity of the protein critically relies on its partnership with Pbx.

Considering the requirement for an intact hexapeptide for the normal activity of Hoxa1, we have addressed here its importance for the oncogenic potential of the protein. Proliferation, anchorage-independent growth and foci assays have been performed to compare the cellular responses to wild-type or hexapeptide mutant Hoxa1. Our data demonstrate that the WM-to-AA substitution in the Hoxa1 hexapeptide severely impairs its oncogenic properties, which therefore suggests the Hoxa1/Pbx partnership to be involved in its ability to transform mammary epithelial cells. Possible implications in terms of therapeutic applications are discussed.

## Results

### The Hoxa1 protein mutated in its hexapeptide has lost the ability to stimulate mammary cells proliferation

Hoxa1 has previously been shown to affect the phenotype of the epithelioid mammary tumor cell line MCF7 in a way that is indicative of its pro-oncogenic activity, as its forced expression enhanced cell proliferation and anchorage-independent growth [29], [35]. To address the importance of the Hoxa1 hexapeptide for its mammary carcinogenic activity, we generated stable MCF7 cell clones for the expression of distinct Hoxa1 variants. The *Hoxa1* gene encodes two alternatively spliced mRNA. A 2.2 kb long mRNA resulting from a single splicing event encodes the full-length protein. A shorter mRNA is obtained when a second, alternative splicing event takes place, generating a frameshift and coding for a truncated protein devoid of homeodomain and hexapeptide sequences [56]. The truncated Hoxa1 variant has been shown to interact with Hoxa1 and Pbx1 and to interfere with the activity of the full-length Hoxa1 [57]. cDNA based expression vectors derived from the long Hoxa1 mRNA could theoretically generate two mRNA species as the alternative splicing event can take place. A first expression vector was designed based on the full length wild-type cDNA (Hoxa1^WT^). A second vector was generated in which the alternative splice site was mutated leading to an Isoleucine-to-Valine substitution at position 115 in the Hoxa1 sequence (Hoxa1^I-V^) which does not affect the Hoxa1 activity in transcription assays [58]. Finally, based on this second construct an expression vector was generated for the Hoxa1 mutant with the core Tryptophan and Methionine residues of the hexapeptide substituted for Alanines (Hoxa1^WM-AA^).

**Figure 1:**
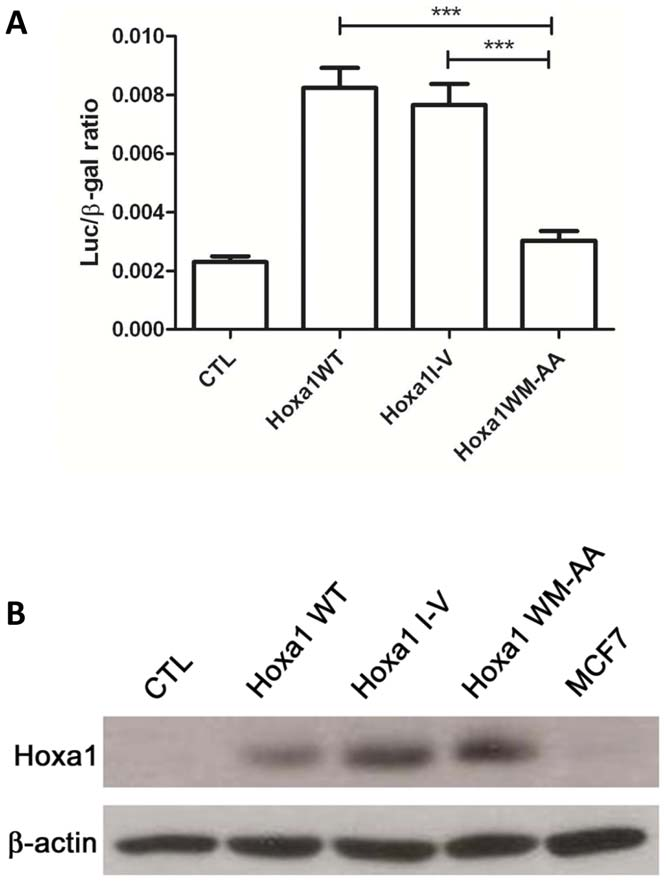
Transcriptional activity and relative expression of Hoxa1 variants. (A) The Hoxa1 target reporter *EphA2-r42B-luc* is activated in MCF7 cells in the presence of expression vectors for Hoxa1^WT^, Hoxa1^I-V^ while not in the presence of Hoxa1^WM-AA^ or of an empty (CTL) plasmid. In each experiment, the pML-EphA2-r42B-luc reporter plasmid was transfected in combination with expression vectors for both Prep1 and Pbx1a. Results were calculated by a luciferase/β-galactosidase ratio and represented as means ± S.D. of triplicates. ***, p<0.001 (ANOVA2). (B) Detection of Hoxa1 variant proteins from whole cell lysates obtained from transiently transfected MCF7 cells reveal that Hoxa1^WT^, Hoxa1^I-V^ and Hoxa1^WM-AA^ proteins are equally expressed and stable. No Hoxa1 protein could be detected from MCF7 cells or from cells transfected with an empty (CTL) expression vector. Detection of constitutively expressed β-actin protein was performed as control load.

To control the relative activity of the variant Hoxa1 proteins, transient co-transfection experiments were first carried out involving a luciferase reporter construct as well as expression vectors for both Pbx1a and Prep1. The pML-EphA2-r42B-luc reporter plasmid contains a cognate Hoxa1 target enhancer derived from the *EphA2* gene, a well-known mammary oncogene. Prep1 is a TALE homeodomain protein which stimulates the nuclear entry of Pbx and which enhances the ability of Hox-Pbx complexes to activate transcription [38]. Cotransfection experiments revealed that the *EphA2-r42B*-*luc* reporter was significantly activated in MCF7 cells expressing Hoxa1^WT^ and Hoxa1^I-V^ proteins, but not in Hoxa1^WM-AA^ expressing cells (Figure 1A). To exclude that the loss of transcriptional activation ability observed for Hoxa1^WM-AA^ was due to a loss in protein stability, the relative abundance of Hoxa1 proteins in transfected cells was evaluated by western blots. Although these western blots are not quantitative, it clearly appeared that the Hoxa1^WM-AA^ was properly expressed, at a similar level as the Hoxa1^WT^ and Hoxa1^I-V^ variants (Figure 1B).

**Figure 2:**
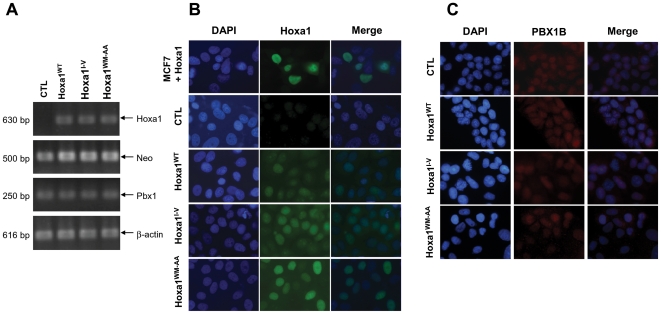
Characterization of MCF7 clones for the constitutive expression of Hoxa1 variants. (A) Expression of Hoxa1, Neomycin resistance (Neo), Pbx1 and β-actin genes was detected by RT-PCR. While MCF7 cells do not express Hoxa1, clones obtained the stable transfection of Hoxa1^WT^, Hoxa1^I-V^ and Hoxa1^WM-AA^ coding plasmids express the Hoxa1 variants at similar levels (β-actin used as reference). All cells express the endogenous Pbx1 gene. (B) The Hoxa1 and (C) PBX1B protein immunolocalisation reveals that both proteins localize into the cell nucleus.

Two stable MCF7 clones were obtained for each expression vector in addition to control clones transfected with the empty vector (CTL). Hoxa1 expression in the selected clones was verified by RT-PCR. (Figure 2A). One amplified fragment corresponding to the full length Hoxa1 mRNA was detected in the MCF7 clones for the Hoxa1^WT^,-Hoxa1^I-V^ and -Hoxa1^WM-AA^ vectors (630 bp, Figure 2A), while no Hoxa1 expression was detected in CTL clones or non-transfected MCF7 cells (not shown). In addition, the fragment expected for the short length Hoxa1 mRNA was never detected in the MCF7-Hoxa1^WT^ cells, suggesting that the alternative splicing does not take place and that the truncated Hoxa1 is not expressed. Therefore, the MCF7 clones for the Hoxa1^WT^ and Hoxa1^I-V^ constructs both express only the full length protein and only differ by the fact that the Hoxa1^I-V^ clones express a single amino-acid variant of Hoxa1. Finally, we also verified that the PBX1 gene is endogenously expressed in all cell clones (Figure 2A), so that in all clones the Hoxa1 protein can potentially interact with its cofactor. Quantitative RT-PCR confirmed that Hoxa1 expression level is not significantly different between the Hoxa1 clones ensuring that cell phenotype changes which could be observed are not due to differences in Hoxa1 expression (data not shown).

To check that the constitutively expressed Hoxa1 variants appropriately reach the cell nucleus to achieve gene regulatory roles, immuno-cytochemical assays were performed (Figure 2B). As expected, the CTL clones did not show Hoxa1 expression. As a positive control, transiently transfected MCF7 cells displayed a strong signal for Hoxa1 in cell nuclei. Nuclear staining of Hoxa1 was detected in all stable clones (Figure 2B). Immuno-cytodetection assay revealed that the endogenously expressed PBX1 protein was the PBX1B isoform and that it also localized into the nucleus of the MCF7 cells and stably transfected derivatives (Figure 2C and data not shown).

**Figure 3:**
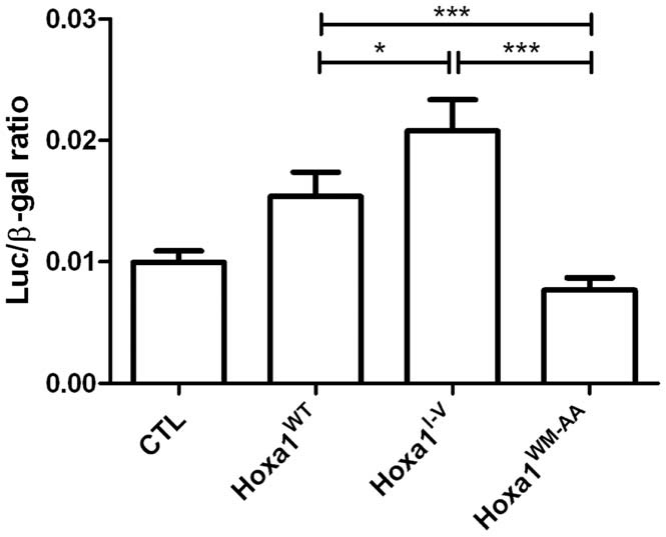
Activation of a Hoxa1 target reporter in mammary carcinoma cell clones. The Hoxa1 target reporter *EphA2-r42B-luc* is activated in cell clones for Hoxa1^WT^ and Hoxa1^I-V^ while not in Hoxa1^WM-AA^ clones. In each experiment, the pML-EphA2-r42B-luc reporter plasmid was transfected in combination with expression vectors for both Prep1 and Pbx1a. The constitutively active pCMV-LacZ reporter plasmid was added as a transfection control. Results were calculated by a luciferase/β-galactosidase ratio, pooled for each type of clones and represented as means ± S.D. of triplicates. *, p<0.05 and ***, p<0.001 (ANOVA 2).

To evaluate if the Hoxa1 variants expressed in the stably transfected clones are transcriptionally active, the pML-EphA2-r42B-luc reporter construct was transiently co-transfected in the stable clones in combination with expression vectors for both Pbx1a and Prep1. Cotransfection experiments revealed that the *EphA2-r42B*-*luc* reporter was significantly activated in the clones expressing Hoxa1^WT^ and Hoxa1^I-V^ proteins, but not in Hoxa1^WM-AA^ expressing clones (Figure 3). That the Hoxa1^WM-AA^ variant was unable to activate the target reporter was confirmed by transient transfection which allows a strong overexpression of the protein [54] (data not shown). These results therefore confirm that MCF7-Hoxa1^WT^ and MCF7-Hoxa1^I-V^ clones express active Hoxa1 proteins, whereas the Hoxa1^WM-AA^ variant has lost the ability to transactivate target genes.

**Figure 4:**
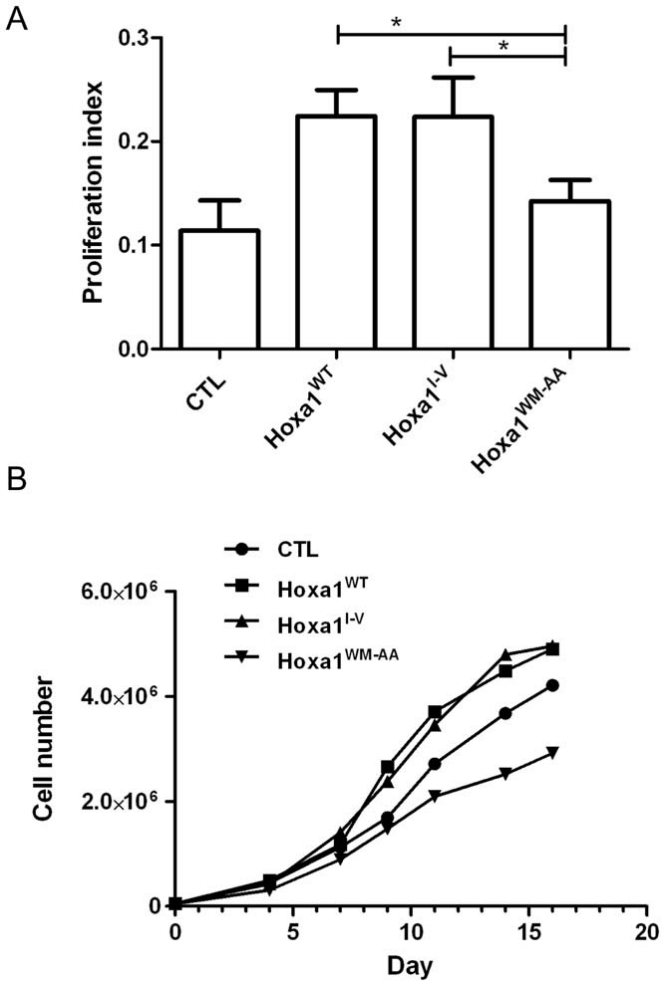
The expression of Hoxa1^WM-AA^ in human mammary carcinoma cells does not result in increased cell proliferation and growth. (A) WST-1 based proliferation assays were performed for MCF7-Hoxa1^WT^, MCF7-Hoxa1^I-V^, MCF7-Hoxa1^WM-AA^ and MCF7-CTL clones. The proliferation index was determined for each clone as described in Materials and Methods. Results were pooled for each type of clones and represented as means ± S.D. of triplicates. *, p<0.05 (ANOVA 2). (B) Cells for MCF7-Hoxa1^WT^, MCF7-Hoxa1^I-V^, MCF7-Hoxa1^WM-AA^ and MCF7-CTL clones were inoculated, kept in culture for 16 days and counted after day 4, 7, 9, 11, 14 and 16. Growth curves represent the mean of four independent experiments.

We then addressed the effect of the hexapeptide substitution on cell growth stimulation provided by Hoxa1. Cell proliferation rate was twice higher for the MCF7-Hoxa1^WT^ and MCF7-Hoxa1^I-V^ clones than for control clones or clones expressing Hoxa1^WM-AA^ mutant (Figure 4A). Interestingly, clones transfected for Hoxa1^WM-AA^ grew at the same rate as the control cells transfected with the empty vector. Complementary to proliferation assays, cell growth was recorded over two weeks of culture, with cell counting after 4, 7, 9, 11, 14 and 16 days of culture. This experiment confirmed that clones expressing the Hoxa1^WT^ and Hoxa1^I-V^ proteins grew twice faster than cells transfected for the Hoxa1^WM-AA^ mutant (Figure 4B). Cells expressing Hoxa1^WM-AA^ however grew slower than the controls, suggesting that this mutant Hoxa1 could exert a dominant negative effect in this cell growth assay (see Discussion). Together these data confirm that the Hoxa1 protein stimulates mammary cell proliferation and that this growth stimulation effect is abrogated by the hexapeptide mutation.

### Anchorage independent cell growth is provided by the wild-type Hoxa1 while not by the hexapeptide mutant

**Figure 5:**
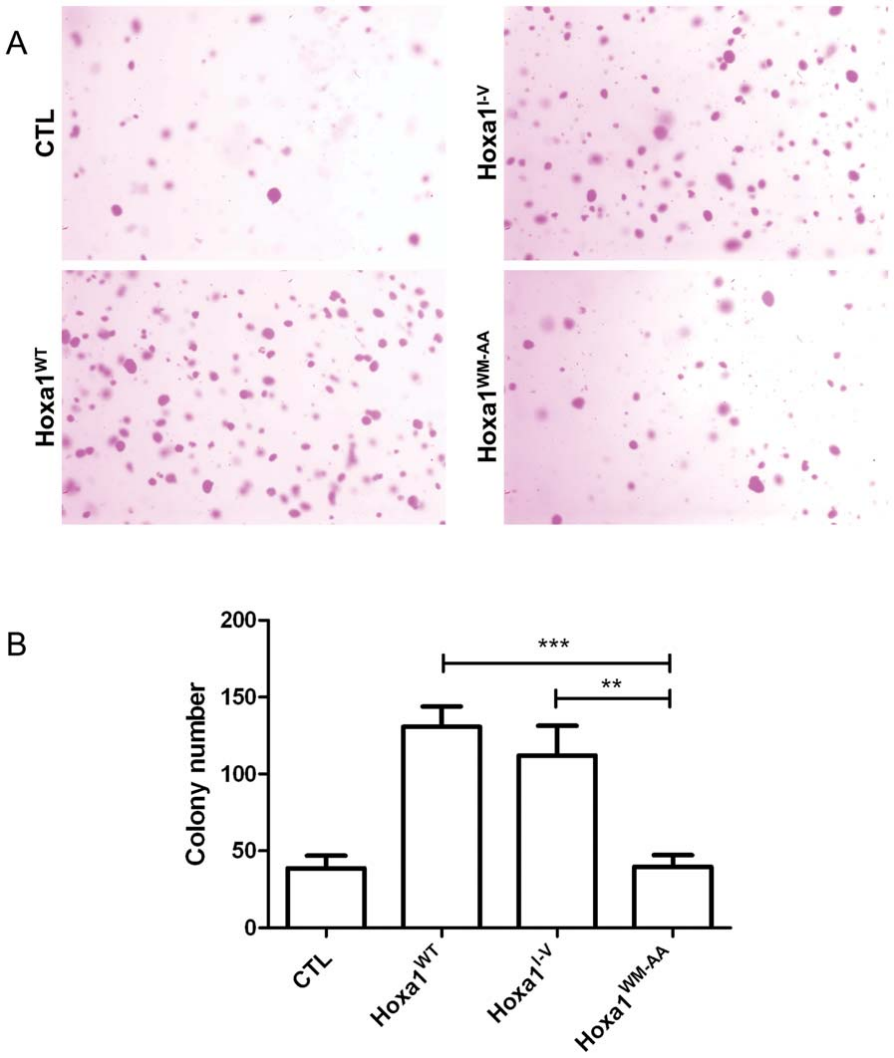
The expression of Hoxa1^WM-AA^ in human mammary carcinoma cells does not result in increased anchorage independent cell growth. Cells were grown in soft agar and colonies were revealed by crystal violet staining (A) MCF7-Hoxa1^WT^ and MCF7-Hoxa1^I-V^ cells produced a lot of colonies in soft agar while CTL and MCF7-Hoxa1^WM-AA^ only provide a modest colony growth. (B) For each culture, colonies were counted in three random microscopic fields at 16X magnification. Results were pooled for each type of clones and represented as means ± S.D of triplicates. **, p<0.01; ***, p<0.001.

Tumor formation is associated with anchorage independent cell growth. This propensity of cells to grow with loose substrate attachment can be assayed in soft-agar medium. Cell suspensions are mixed in low percentage agar and left for growing over 17 days. Cells able to grow in an anchorage-independent manner will form colonies easily viewed after crystal violet staining. Cell clones were grown in soft agar and colonies were counted after 17 days of culture. As depicted on Figure 5, a low number of colonies were formed by the CTL cells. In contrast about three times more colonies grew from the Hoxa1^WT^ and Hoxa1^I-V^ expressing clones. Finally, the Hoxa1^WM-AA^ clones produced a similar amount of colonies as the control clones, demonstrating that the mutant Hoxa1 protein has lost its ability to promote anchorage-independent cell growth (Figure 5).

### Hoxa1^WM-AA^ expressing cells show contact inhibition

**Figure 6:**
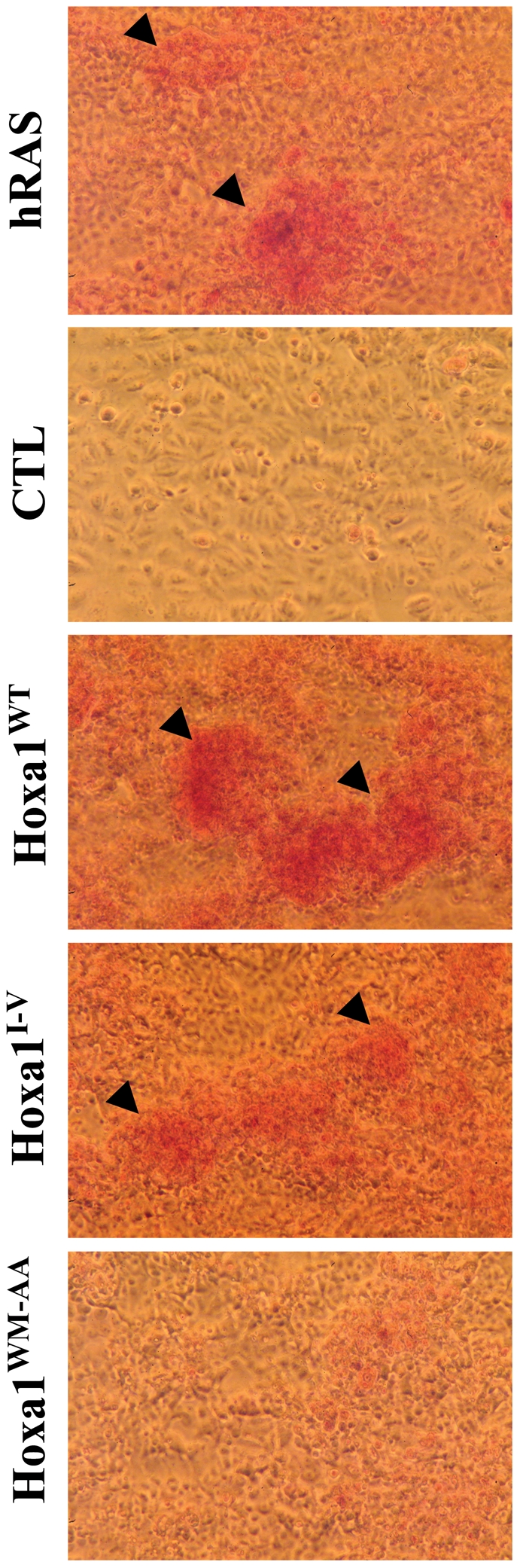
Hoxa1^WT^ and Hoxa1^I-V^ relieve MCF7 cells from contact inhibition, while expressing Hoxa1^WM-AA^ does not. MCF7 cells were transiently transfected for Hoxa1^WT^, Hoxa1^I-V^ and Hoxa1^WM-AA^, together with Pbx1a and Prep1 cofactors, and grown for three weeks. Controls included cells transfected for the potent oncogene hRAS or cells transfected for Pbx1a and Prep1 only. Foci formation was observed for hRAS, Hoxa1^WT^ and Hoxa1^I-V^ transfected cells (arrowheads) while not for CTL and Hoxa1^WM-AA^ cells.

Tumor cells loose the contact inhibition normally observed for epithelial cells *in vivo* or *in vitro* when cells reach confluence. The loss of contact inhibition induced by oncogenes is classically monitored by a foci formation assay. In this assay, cells are transiently transfected to express oncoproteins and are left to grow for three weeks. Untransfected MCF7 cells displaying an epithelioïd phenotype are responsive to contact inhibition and show very few, if any, foci after three weeks of culture. The transient transfection of Prep1a and Pbx1 expression vectors in control clones did not enhance foci formation (CTL, Figure 6). In contrast, transfecting Hoxa1^WT^ or Hoxa1^I-V^ together with the cofactors resulted in the appearance of numerous foci (Figure 5). Most significantly, when the hexapeptide mutant was cotransfected with the cofactors, a small amount of foci was observed, which was not distinguishable from the situation where only the cofactors were expressed. This assay therefore shows that the Hoxa1^WM-AA^ protein has lost the ability to relieve the cells from their contact inhibition. This again supports that the hexapeptide mutation suppresses the oncogenic potential of Hoxa1.

## Discussion

While a continuously increasing number of studies report correlations between *Hox* genes misexpression and several types of cancers, only a few *Hox* genes have been identified to actually impact on cancer progression, as genuine oncogenes or tumor suppressors [11], [13]. *Hoxa1* has been reported to be abnormally expressed in breast carcinomas [8], [25] and to act as a mammary oncogene [29]. Like many other Hox proteins, Hoxa1 can interact with the TALE homeoproteins Pbx. This interaction relies on a hexapeptidic motif of Hoxa1. It has indeed been demonstrated that substituting two amino acids (WM to AA) in this hexapeptide motif abrogated the formation of Hoxa1-Pbx1a complexes on cognate target DNA sequences [59], [60]. Further, we have previously shown that disrupting the Hoxa1-Pbx interaction severely impaired its developmental activity. Indeed, by substituting these two amino acids (WM to AA) critically involved in the docking to Pbx, we generated knockin mice which phenocopied the Hoxa1 knockout, suggesting that the Hoxa1-Pbx partnership is crucial to the Hoxa1 function. [55]

Here, we addressed the importance of the hexapeptide integrity for the oncogenic potential of Hoxa1. We demonstrate that the Hoxa1^WM-AA^ hexapeptide mutant lost its ability to stimulate cell proliferation, anchorage-independent cell growth and loss of contact inhibition. Thus, this hexapeptide motif is required to confer to Hoxa1 its oncogenic potential, supporting the view that this critically relies on the ability of Hoxa1 to interact with Pbx.

The involvement of Hox-Pbx interaction in cancer stimulation is supported by several studies aiming at evaluating the impact of HOX-PBX dimer disrupting molecules on cancer cell properties. These molecules were either synthetic peptides mimicking the hexapeptide motif from HOX proteins [61], [62], [63], [64], or mimetic compounds obtained from molecular modelling and combinatorial libraries [65]. Such antagonist molecules have been shown to specifically block proliferation and promote apoptosis of melanoma, ovarian, pancreatic and non-small-cell lung cancer cells in which members of the HOX family are deregulated [61], [63]. Blocking the activity of HOX protein by interfering with their binding to PBX co-factor also reduced the growth of tumor cells *in vivo* [61], [63]. The cell behavior modifications induced by these inhibitors of the HOX-PBX interaction were further correlated to transcriptional changes indicative of a loss of malignancy [61], [62], [63]. In a similar approach, Fernandez et al. [66] showed that a dominant negative mutant of PBX reduced the oncogenic activity of HoxB7 and correlated well with increased apoptosis and decreased cell cycling. Finally, mutating the hexapeptide of HOXB4 has also shown to impair its ability to provoke cell transformation [67]. All these studies together with our data suggest that the interaction between Hox and Pbx proteins is a potential therapeutic target for distinct types of cancers.

Nevertheless, disrupting the Hox-Pbx interaction could not always result in a simple functional invalidation of the Hox activity. Indeed, a double mutation in the hexapeptide motif of the mouse Hoxb8 did not result in a loss-of-function of the protein as it is shown here for Hoxa1 and as we previously showed for the Hoxa1^WM-AA^ knockin mice [55], [68]. The knockin allele of *Hoxb8* coding for a hexapeptide mutant protein indeed appeared as a neomorph. Thus, in contrast to what stands for Hoxa1, the hexapeptide-mediated interaction with Pbx would rather have a modulatory implication on the activity of Hoxb8. The use of hexapeptide mimetic peptides or of related molecules in a therapeutical perspective should then be considered on a case-by-case basis [68] and it would be worth addressing the functional importance of the hexapeptide for additional Hox proteins involved in cancer stimulation.

Although the integrity of the hexapeptide is required for the oncogenic activity of Hoxa1, this does not necessarily imply that the Hoxa1-Pbx interaction is involved in the Hoxa1-mediated oncogenesis. We cannot formally exclude that the loss of oncogenic potential due to the hexapeptide mutation is independent of the loss of Pbx interaction. Indeed, the hexapeptide might be involved in other critical interactions as has been shown for other Hox proteins. For example, study of the hexapeptide motif of Antennapedia, a Hox protein from drosophila, has revealed that it is involved in an interaction with a TATA-binding associated factor linking Antennapedia to the transcripitonal machinery [69]. However, hexapeptide-mediated interactions with other proteins than Pbx have never been reported for Hoxa1, its paralogues or its invertebrate homologues.

Intriguingly, while Hoxa1 expression stimulated cell growth, expression of the Hoxa1^WM-AA^ variant resulted in a decrease in cell growth with respect to control cells in one of our assays. This suggests that beside the loss of transcription activity and Pbx interaction displayed by Hoxa1^WM-AA^, this variant could exert a dominant negative effect towards proteins involved in cell proliferation. It is highly expectable that Hoxa1 is involved in diverse protein-protein interactions other than with the sole TALE transcription factors. The WM-AA substitution would not invalidate all those interactions so that although being inactive in mediating Hoxa1-Pbx dimer formation on DNA, this mutant still interacting with other factors to be identified could impair the activity of some of those interactors thereby acting as a dominant negative.

The present study identifies the hexapeptide as a key determinant of Hoxa1 oncogenic properties. Considering the growing body of evidence that Hox proteins can be critical actors in several kinds of cancers, deciphering the modalities of their oncogenic or oncosuppressive activities will undoubtedly be relevant for the clinic and future therapeutic developments.

## Materials and Methods

### Plasmid constructions

Expression vectors for Hoxa1 derivatives were obtained from the previously described pGIH309, pGIH327 and pGIH328 constructs [54]. Shortly, pGIH309 bears the wild-type Hoxa1 cDNA (Hoxa1^WT^) under the control of a CMV enhancer/promoter module. pGIH327 is similar to pGIH309 but harbours a mutant Hoxa1 cDNA in which an alternative splice site has been mutated (Hoxa1^I-V^) which in turn results in an I-to-V amino acid substitution in the Hoxa1 protein [58]. pGIH328 also contains a Hoxa1 cDNA sequence invalidated for the alternate splicing and is additionally modified to code for the WM-to-AA substitution in the Hoxa1 hexapeptide (Hoxa1^WM-AA^). To allow selecting stably transfected cells for these expression vectors, a Neomycin resistance marker has been added in pGIH309, pGIH327 and pGIH328 to give rise to pGIH364, pGIH367 and pGIH368, respectively. An empty vector only coding for Neomycin resistance has been obtained as control for all the experiments (pNeo). Details regarding the plasmid constructs are available upon request. Reporter plasmids EphA2-r4-Luc [70] and pCMV-LacZ [71], as well as expression vectors for Pbx1a [54] and Prep1 [72] have been described elsewhere.

### Cell culture and transfections

The MCF7 cell line (ATCC #HTB-22) and transfected derivatives were maintained at 37°C in a humidified, 5% CO_2_ atmosphere in DMEM 4.5 g/L D-glucose supplemented with 10% heat-inactivated fetal bovine serum (FBS), 100 IU/ml penicillin and 100 µg/ml streptomycin and 2 mM L-glutamine (Gibco). MCF7 cells were stably transfected with pNeo, pGI364, pGIH367 and pGIH368 plasmids, by use of the Gene Pulser Xcell System (Bio Rad). Transfectants were selected in 1 mg/ml G418 (Gibco). Transient co-transfections for luciferase reporter assays were carried out with the Transfectin reagent (BioRad). One day prior to transfection 80 000 cells per well were seeded in 24-well plates. Each transfection involved a total amount of 1.05 µg of DNA, containing: 0.625 µg of reporter plasmid (pML-EphA2-r42B-luc); 0.125 µg of Hox expression vector; 0.125 µg of Pbx1a expression vector; 0.125 µg of Prep1 expression vector; and 0.05 µg of internal standard reporter plasmid (pCMV-LacZ). In co-transfections aimed at detecting foci formation, 200 000 cells were seeded in 36-mm Petri culture dishes. They have been transfected after 24 hours with 1 µg of Hoxa1 or control expression vector and 1 µg of each of the Pbx1a and Prep1 expression vectors with the Transfectin reagent (BioRad). As positive control, a plasmid coding for the oncogene hRAS, was used.

### Western blotting

For detection of Hoxa1 proteins expression, transiently transfected cells were lysed with RIPA buffer (1 mM EDTA, 0.1% SDS, 1% Nonidet P40, 0.5% NaDeoxycholate, 50 mM TrisHCl pH7.5, 250 mM NaCl, protease inhibitors). Whole cell lysates were run on a SDS-PAGE, blotted on a nitrocellulose membrane and revealed with an anti-Hoxa1 rabbit antibody (1/500; Sigma HPA004933), anti-rabbit bovine IgG-horseradish peroxidase (HRP)-conjugated antibody (1/3000; SantaCruz sc-2379). The protein load for western blotting was controlled by detecting β-actin with a HRP conjugated anti-β-actin antibody (1/3000 Sigma A3854).

### Reverse transcriptase-PCR

Total RNA was extracted using Trizol Reagent (Invitrogen). RNA quantification was performed on a Nanodrop apparatus (Thermo Scientific). One µg of RNA was then reverse transcribed into cDNA and amplified with the Expand Reverse Transcriptase (Roche) and Taq Polymerase (Westburg) respectively. Primers for RT-PCR were as follows: *Hoxa1* (forward), 5′-CCTTATGGCCCCTATGGA-3′; *Hoxa1* (reverse), 5′-TTCTCAGATGATTCTTCCGTT-3′; *β-actin* (forward), 5′-GCTGGAAGGTGGACAGCGA-3′; *β-actin* (reverse), 5′-GGCATCGTGATGGACTCCG-3′; *Neo^R^* (forward), 5′-AATGAACTGCAGGACGAGGC-3′; *Neo^R^* (reverse), 5′- CAACGCTATGTCCTGATAGC-3′; *Pbx1* (forward), 5′-TCAGAGATGGATGCGAGGGCGAAGAGACGC-3′; *Pbx1* (reverse), 5′-TTTGGCAGCATAAATATTGGC-3′. All RNA samples were treated with DNase I to avoid genomic DNA contamination.

### Immunostaining and fluorescence microscopy

Immunodetections of Hoxa1 and PBX1B were performed either on stably or on transiently transfected cells. In both cases, cells were seeded on glass cover-slips in 24-well plates. For stable clones, twenty-hours after seeding, cells were fixed in 4% formalin and blocked in 10% powder milk. Cells were incubated at 4°C with the anti-Hoxa1 rabbit antibody (1/50, Sigma HPA004933) or anti-Pbx1B (41.1) mouse antibody (1/50, Santacruz sc-101852) overnight. They were washed and incubated respectively with a fluorescein coupled anti-rabbit IgG antibody (1/100, GE Healthcare N1034) or with an Alexa Fluor 555 coupled anti-mouse IgG antibody (1/1000, Cell Signaling 4409) for 1 h. Cover-slips were mounted in vectashield with DAPI medium (Vector Laboratories H1200) and viewed under Polyvar microscope (Reichert Jung). For transiently transfected cells, the same procedure was applied, except that cells were firstly transfected 24 hours after seeding and then processed for immunostaining 24 hours after transfection.

### Luciferase reporter assay

Cells were harvested 48 h after transfection for enzymatic assays. Lysis and enzymatic activity dosages were performed with the β-gal Reporter Gene Assay (Chemiluminescent) kit (Roche) and the Luciferase Reporter Gene Assay (High Sensitivity) kit (Roche), according to the instructions provided by the manufacturer. For each transfection, the constitutively active pCMV-LacZ reporter was used as a control, so that the relative luciferase activity was calculated by a luciferase/β-galactosidase ratio.

### Cell proliferation and growth assays

The WST-1 assay is a colorimetric method based on the cleavage by mitochondrial dehydrogenase of the tetrazolium salt generating a detectable product, formazan. Two thousand cells for each MCF7 clone were seeded in 96-well plates in complete DMEM, with 2 wells devoid of cells as blank samples. Cells were allowed to seed overnight at 37°C in 5% CO2. Twenty-four hours after seeding, medium was changed with DMEM supplemented with 1% FBS. Two days after seeding, 10 µl of WST-1 reagent was added to the cells medium for 4 hours. The absorbance for the formazan product (440 nm) and the background control (620 nm) were recorded every hour by a multiplate reader (SpectraMax 190, Molecular Devices). For each time point, the difference between the measures at 440 nm and 620 nm was calculated and inserted on a graph as a function of time. The slope of each curve was calculated and represented the cell proliferation index.

For growth recording, 5.0 10^4^ cells of each MCF7 clone were seeded in 6-well plates in complete DMEM. Medium was changed every 2 days. After 4, 7, 9, 11, 14 and 16 days of culture, cells were counted. Values were reported on a graph representing the cell growth of MCF7 clones.

### Anchorage-independent growth assay

Anchorage-independent growth was assayed in soft agar. Cells were plated into 24-well plates in growth medium (DMEM) containing 0.3% agarose, on top of a layer of 0.6% agarose gel (Sigma A9045). After 17 days, cells were stained with crystal violet for 1 h and colonies were counted under a binocular (Wild M3B – Van Hopplynus Instrument) in three random microscopic fields at 16X magnification.

### Foci formation assay

MCF7 cells were seeded in 36-mm Petri dish and co-transfected as mentioned above. Once confluence was reached, medium was changed every 3 days. After 2 weeks, cultures were fixed with formalin, stained with 1% rhodamine B (Sigma R6626) and washed with PBS to bleach the non-focal monolayer. Foci were observed under a binocular (Leitz Wetzlar).
